# Supplementary material for: The gut commensal fungus, Candida parapsilosis, promotes high fat-diet induced obesity in mice
Source: Commun Biol. 2021 Oct 25;4:1220. doi: 10.1038/s42003-021-02753-3 (PMC8546080; doi:10.1038/s42003-021-02753-3)
Supplement: Supplementary file 2 — Supplementary Information [file 42003_2021_2753_MOESM2_ESM.pdf]

The gut commensal fungus, *Candida parapsilosis*, promotes high fat-diet induced obesity in mice

Shanshan Sun[1, 2, 3, #], Li Sun[1,4, #], Kai Wang[1, #], Shanshan Qiao[1,4], Xinyue Zhao[5], Xiaomin Hu[6], Wei Chen[7], Shuyang Zhang[5], Hantian Li[1], Huanqin Dai[1,4*], Hongwei Liu[1,4*]

1 State Key Laboratory of Mycology, Institute of Microbiology, Chinese Academy of Sciences, Beijing 100101, China

2 School of Life Sciences, University of Science and Technology of China, Hefei, China

3 The Second Hospital of Anhui Medical University, Hefei, China

4 University of Chinese Academy of Sciences, Beijing, 100049, China

5 Department of Cardiology, Peking Union Medical College Hospital, Chinese Academy of Medical Science & Peking Union Medical College, Beijing, 100730, China

6 Department of Medical Research Center, State Key Laboratory of Complex Severe and Rare Diseases, Peking Union Medical College Hospital, Chinese Academy of Medical Science & Peking Union Medical College, Beijing, 100730, China

7 Department of Clinical Nutrition, Dept. of Health Medicine, Peking Union Medical College Hospital, Chinese Academy of Medical Sciences and Peking Union Medical College, Beijing, China

* Corresponding Author Tel: +86 10 64806074; E-mail: liuhw@im.ac.cn (H-W, Liu) or daihq@im.ac.cn (Huanqin Dai)

[#] SS Sun, L Sun and K Wang contributed equally to this work.

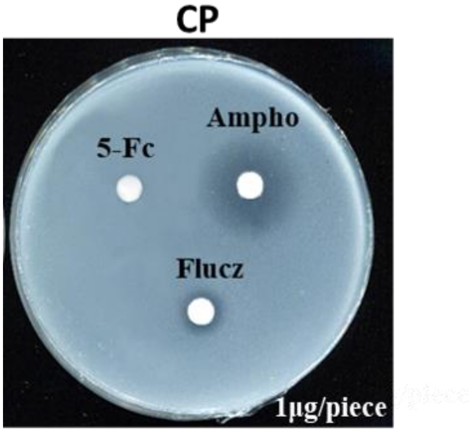

Supplementary Figure 1. Antifungal effect of amphotericin B, fluconazole, and 5-fluorocytosine on *C. parapsilosis*. The *C. parapsilosis* was cultured on PDA along with 1 μg of amphotericin B, fluconazole, and 5-fluorocytosine in each paper disc.

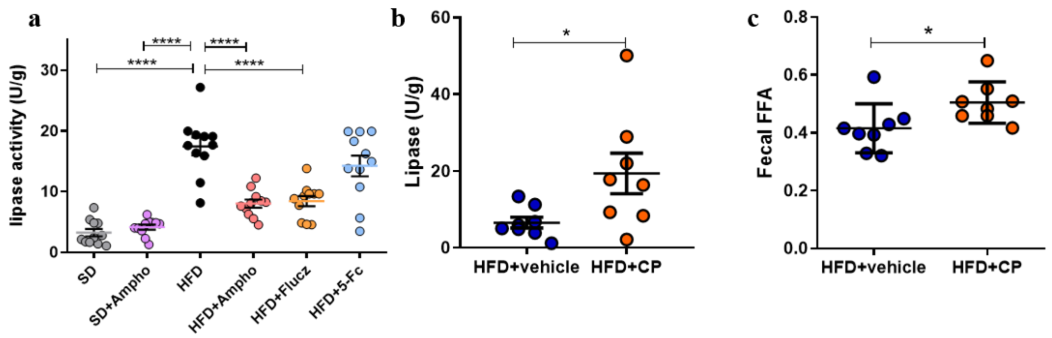

Supplementary Figure 2. The levels of lipase activity, FFA under the antifungal treatment and the oral treatment with *C. parapsilosis*. (a) Fecal lipase activity after antifungal treatment; (b) Fecal lipase activity after intragastric administration of *C. parapsilosis*; (c) Fecal FFA after intragastric administration of *C. parapsilosis*. SD, stand diet fed mice group; SD+Ampho, stand diet fed mice treated with amphotericin B; HFD, high-fat diet fed mice group; HFD+Ampho, high-fat diet fed mice treated with amphotericin B; HFD+Flucz, high-fat diet fed mice treated with fluconazole; HFD+5-Fc, high-fat diet fed mice treated with 5-fluorocytosine. HFD+vehicle, high-fat diet fed and fungi-free mice treated with PBS; HFD+CP, high-fat diet fed and fungi-free mice treated with the live *C. parapsilosis*. Data are presented as the mean ± SEM. (a) N = 11mice per group (b-c) N = 8 mice per group. Statistical analysis was performed using one-way ANOVA followed by the Tukey post hoc test for (a-c). *p < 0.05; **p < 0.01; ***p < 0.001.

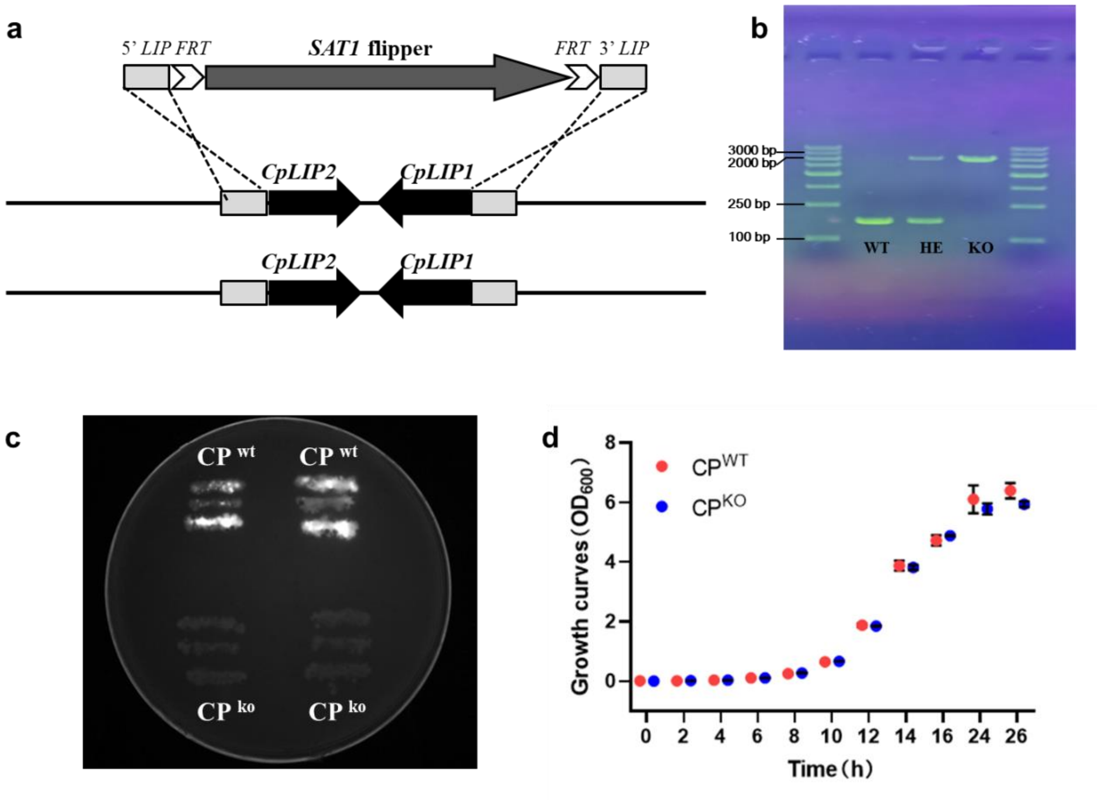

Supplementary Figure 3. Construction of lipase mutant strain of *C. parapsilosis*. (a, b) Design of gene targeting for the *C. parapsilosis* lipase locus showing the SAT1 flipper cassette with the homologous *C. parapsilosis* lipase fragments 5′ *LIP* and 3′ *LIP* as well as the FLP recombination target sequences (FRT). (c) Lipolytic activity of *C. parapsilosis* (CP^{WT}) and lipase mutants (CP^{KO}) presented on 90mm petrie dish; (d) Growth curves of *C. parapsilosis* (CP^{WT}) and lipase-negative mutant strain of *C. parapsilosis* (CP^{KO}) in YPD media. Statistical analysis was performed using two-way ANOVA followed by the Bonferroni post hoc test for (d).

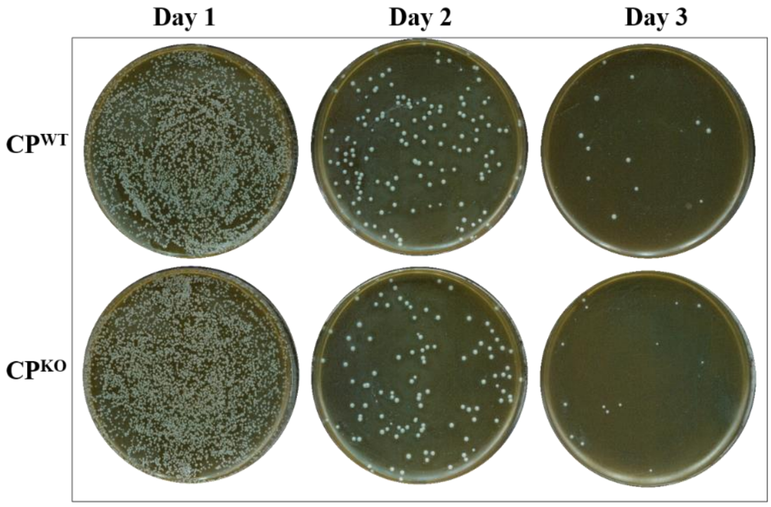

Supplementary Figure 4. Colonization of the wild type strain (CP^{WT}) and the lipase-negative mutant strain of *C. parapsilosis* (CP^{KO}) in the colon of HFD-fed mice. Feces were obtained and cultured on days 1–3 after oral administration of the wild type strain and the lipase-negative mutant strain of *C. parapsilosis* in the amphotericin B-pretreated HFD-fed mice, respectively.

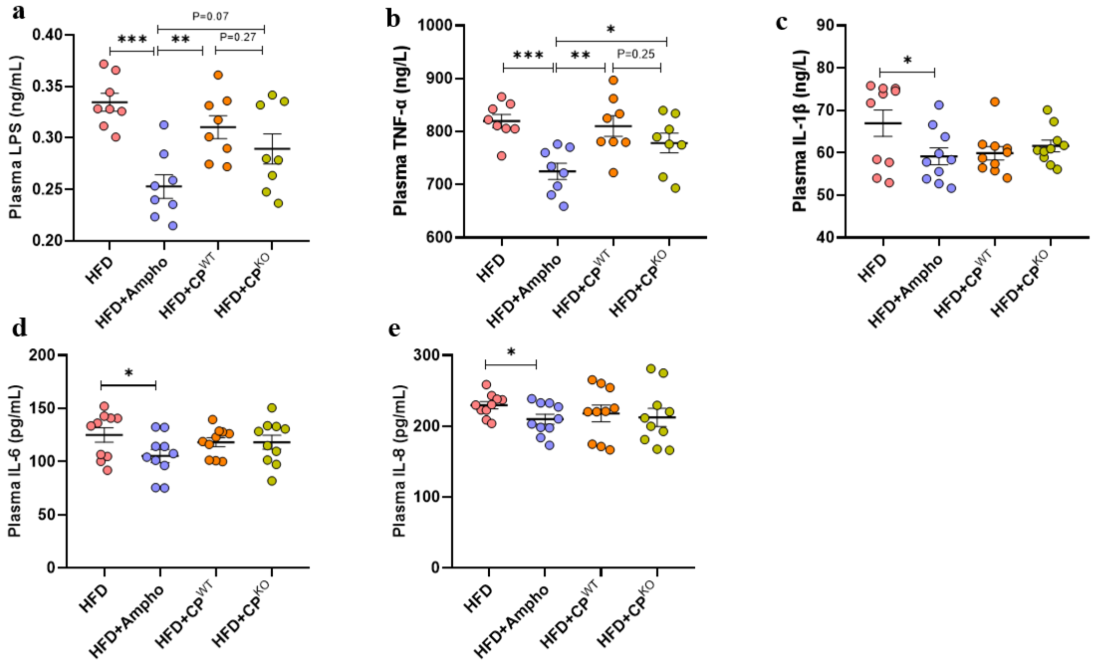

Supplementary Figure 5. Effects of the wild type strain and the lipase mutant strain of *C. parapsilosis* on inflammation in HFD-induced obese mice. The HFD-fed mice were pretreated by addition of AmpB in the drinking water for two weeks, and then orally given with the live *C. parapsilosis* (CP^{WT}) or the lipase-negative mutant strain of *C. parapsilosis* (CP^{KO}) for 7 weeks. (a) plasma LPS; (b) plasma TNF-α; (c) plasma IL-1β; (d) plasma IL-6; (e) plasma IL-8; (f) relative mRNA expression of CD36 in liver. DIO, high-fat diet fed mice; HFD+Ampho, high-fat diet fed mice treated with amphotericin B; HFD+CP^{WT}, high-fat diet fed and fungi-free mice treated with live *C. parapsilosis*; HFD+CP^{KO}, high-fat diet fed and fungi-free mice treated with the lipase-negative mutant strain of *C. parapsilosis*. Data are presented as the mean ± SEM. N = 7-10 mice per group. Statistical analysis was performed using one-way ANOVA followed by the Tukey post hoc test (A-E). $^{*}p < 0.05$; $^{**}p < 0.01$; $^{***}p < 0.001$.

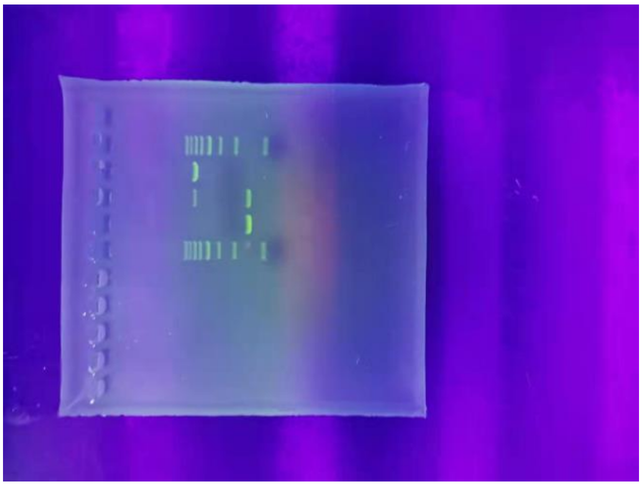

Supplementary Figure 6. Uncropped versions of Supplementary Figure 3b

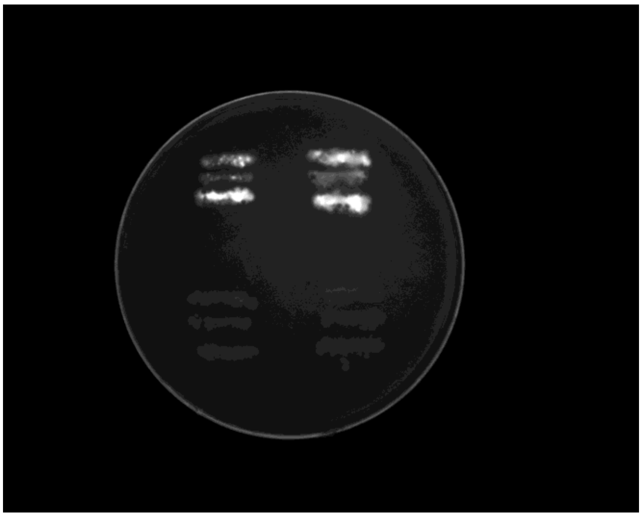

Supplementary Figure 7. Uncropped versions of Supplementary Figure 3c

Supplementary Table 1. Minimum inhibitory concentration (MIC_{80}) values ([μg/mL]) of amphotericin B and fluconazole on *C. parapsilosis*.

Drug	CP
amphotericin B	6.25±0.25
fluconazole	12.5±0.55

Supplementary Table 2. Main characteristics of the samples used in this study.

Sample name	Collection date	Clinical status	Age	Sex	BMI
health 1	2021	Health	32	Male	21.4
health 2	2021	Health	35	Male	23.8
health 3	2021	Health	57	Male	21.1
health 4	2021	Health	33	Female	20.9
health 5	2021	Health	33	Female	23.4
health 6	2021	Health	35	Female	21.8
health 7	2021	Health	36	Male	21.7
health 8	2021	Health	42	Male	18.2
health 9	2021	Health	57	Male	24
obesity 1	2021	Obesity	38	Male	39.2
obesity 2	2021	Obesity	20	Male	41.9
obesity 3	2021	Obesity	45	Male	34.6
obesity 4	2021	Obesity	29	Male	63.1
obesity 5	2021	Obesity	30	Male	35.5
obesity 6	2021	Obesity	35	Female	33
obesity 7	2021	Obesity	34	Female	31.1
obesity 8	2021	Obesity	28	Female	30.4

Supplementary Table 3. Primer sequences used for the quantitative PCR.

Primer Name	Sequence (5' to 3')
Gapdh-F	AGGTCGGTGTGAACGGATTTG
Gapdh-R	TGTAGACCATGTAGTTGAGGTCA
CD36-F	TCCTCTGACATTTGCAGGTCTATC
CD36-R	AAAGGCATTGGCTGGAAGAA
ITS1-F	TCCGTAGGTGAACCTGCGG
ITS4-R	TCCTCCGCTTATTGATATGC
Cpar-F	GATCAGACTTGGTATTTTGTATGTTACTCTC
Cpar-R	CAGAGCCACATTTCTTTGCAC
