# Supplementary material for: The gut commensal fungus, Candida parapsilosis, promotes high fat-diet induced obesity in mice
Source: Commun Biol. 2021 Oct 25;4:1220. doi: 10.1038/s42003-021-02753-3 (PMC8546080; doi:10.1038/s42003-021-02753-3)
Supplement: Supplementary file 3 — Description of Additional Supplementary Files [file 42003_2021_2753_MOESM3_ESM.pdf]

Description of Additional Supplementary Files

File name: Supplementary Data 1.
Description: Source data for graphs and charts.
