# Supplementary material for: The gut commensal fungus, Candida parapsilosis, promotes high fat-diet induced obesity in mice
Source: Commun Biol. 2021 Oct 25;4:1220. doi: 10.1038/s42003-021-02753-3 (PMC8546080; doi:10.1038/s42003-021-02753-3)
Supplement: Supplementary file 5 — Reporting Summary [file 42003_2021_2753_MOESM5_ESM.pdf]

nature portfolio

COMMSBIO-21-1875

Corresponding author(s): Hongwei Liu

Last updated by author(s): Sep 23, 2021

Reporting Summary

Nature Portfolio wishes to improve the reproducibility of the work that we publish. This form provides structure for consistency and transparency in reporting. For further information on Nature Portfolio policies, see our Editorial Policies and the Editorial Policy Checklist.

Statistics

For all statistical analyses, confirm that the following items are present in the figure legend, table legend, main text, or Methods section.

n/a	Confirmed	
☐	☒	The exact sample size (*n*) for each experimental group/condition, given as a discrete number and unit of measurement
☐	☒	A statement on whether measurements were taken from distinct samples or whether the same sample was measured repeatedly
☐	☒	The statistical test(s) used AND whether they are one- or two-sided<br>*Only common tests should be described solely by name; describe more complex techniques in the Methods section.*
☐	☒	A description of all covariates tested
☒	☐	A description of any assumptions or corrections, such as tests of normality and adjustment for multiple comparisons
☐	☒	A full description of the statistical parameters including central tendency (e.g. means) or other basic estimates (e.g. regression coefficient) AND variation (e.g. standard deviation) or associated estimates of uncertainty (e.g. confidence intervals)
☐	☒	For null hypothesis testing, the test statistic (e.g. *F*, *t*, *r*) with confidence intervals, effect sizes, degrees of freedom and *P* value noted<br>*Give P values as exact values whenever suitable.*
☒	☐	For Bayesian analysis, information on the choice of priors and Markov chain Monte Carlo settings
☒	☐	For hierarchical and complex designs, identification of the appropriate level for tests and full reporting of outcomes
☒	☐	Estimates of effect sizes (e.g. Cohen's *d*, Pearson's *r*), indicating how they were calculated

Our web collection on statistics for biologists contains articles on many of the points above.

Software and code

Policy information about availability of computer code

Data collection	Data were analyzed using Prism (GraphPad 6.0.) and are presented as mean ± SEM. Statistical significance was determined using the unpaired two-tailed Student's t test for single variables and two-way ANOVA for two variables. A p-value of <0.05 is considered to be statistically significant.
Data analysis	Data were analyzed using Prism (GraphPad 6.0.) and are presented as mean ± SEM. Statistical significance was determined using the unpaired two-tailed Student's t test for single variables and two-way ANOVA for two variables. A p-value of <0.05 is considered to be statistically significant.

For manuscripts utilizing custom algorithms or software that are central to the research but not yet described in published literature, software must be made available to editors and reviewers. We strongly encourage code deposition in a community repository (e.g. GitHub). See the Nature Portfolio guidelines for submitting code & software for further information.

Data

Policy information about availability of data

All manuscripts must include a data availability statement. This statement should provide the following information, where applicable:

- Accession codes, unique identifiers, or web links for publicly available datasets
- A description of any restrictions on data availability
- For clinical datasets or third party data, please ensure that the statement adheres to our policy

The 16S rRNA sequencing data were deposited in the National Microbiology Data Center (Accession numbers: NMDC40004565)

Field-specific reporting

Please select the one below that is the best fit for your research. If you are not sure, read the appropriate sections before making your selection.

☒ Life sciences ☐ Behavioural & social sciences ☐ Ecological, evolutionary & environmental sciences

For a reference copy of the document with all sections, see nature.com/documents/nr-reporting-summary-flat.pdf

Life sciences study design

All studies must disclose on these points even when the disclosure is negative.

Sample size	The sample size of animal experiments was at least 5 or more animals in order to obtain statistical signification. Sample size was estimated on the basis of sample availability and previous studies.
Data exclusions	Data beyond the mean ±3*standard deviation is excluded
Replication	all attempts at replication were successful
Randomization	The body weight of high fat DIO mice was increased to about 20% of the normal chow diet mice. DIO mice were randomly divided on the basis of the body weight and blood glucose
Blinding	Blinding was not possible, because we only need human excrement, human consciousness doesn't affect the results

Reporting for specific materials, systems and methods

We require information from authors about some types of materials, experimental systems and methods used in many studies. Here, indicate whether each material, system or method listed is relevant to your study. If you are not sure if a list item applies to your research, read the appropriate section before selecting a response.

Materials & experimental systems

n/a	Involved in the study	
☒	☐	Antibodies
☒	☐	Eukaryotic cell lines
☒	☐	Palaeontology and archaeology
☐	☒	Animals and other organisms
☐	☒	Human research participants
☒	☐	Clinical data
☒	☐	Dual use research of concern

Methods

n/a	Involved in the study	
☒	☐	ChIP-seq
☒	☐	Flow cytometry
☒	☐	MRI-based neuroimaging

Animals and other organisms

Policy information about studies involving animals; ARRIVE guidelines recommended for reporting animal research

Laboratory animals	8-week-old C57BL/6J male
Wild animals	the study did not involve wild animals
Field-collected samples	Mice were housed in a room with controlled temperature at 20–25 °C and humidity of 40%–60% and a 12 h light/dark cycle with free access to normal water and chow.
Ethics oversight	All procedures were performed in accordance with recommendations under the Guidance for the Care and Use of Laboratory Animals of the Institute of Microbiology, Chinese Academy of Sciences (IMCAS) Ethics Committee.he protocol was approved by the Committee on the Ethics of Animal Experiments of IMCAS (Permit APIMCA2019030).

Note that full information on the approval of the study protocol must also be provided in the manuscript.

Human research participants

Policy information about studies involving human research participants

Population characteristics	The study sample consisted of 17 stool samples, including 9 healthy people and 8 obese people, with detailed information is shown in Supplementary Table 2.
Recruitment	No enrolled subjects were given antibiotic, antifungal, or antiparasitic treatment at the time of sample collection or during

Recruitment	the previous 2 months. Stool samples from each patient were collected and immediately transported to the laboratory in an ice-packed container and stored at -80? for use.
Ethics oversight	Our research was approved by the Ethics Committee of Peking Union Medical College Hospital (PUMCH) and was conducted according to the principles of the Declaration of Helsinki. All individuals who participated in this study provided written informed consent, and the protocol was approved by PUMCH.

Note that full information on the approval of the study protocol must also be provided in the manuscript.
